# Ethics framework for citizen science and public and patient participation in research

**DOI:** 10.1186/s12910-022-00761-4

**Published:** 2022-03-13

**Authors:** Barbara Groot, Tineke Abma

**Affiliations:** Leiden University Medical Center (LUMC), Albinusdreef 2, 2333 ZA Leiden, The Netherlands

**Keywords:** Research ethics, Ethics framework, Ethics work, Citizen science, Patient and public engagement, Medical Research Ethics Committee

## Abstract

**Background:**

Citizen science and models for public participation in health research share normative ideals of participation, inclusion, and public and patient engagement. Academic researchers collaborate in research with members of the public involved in an issue, maximizing all involved assets, competencies, and knowledge. In citizen science new ethical issues arise, such as who decides, who participates, who is excluded, what it means to share power equally, or whose knowledge counts. This article aims to present an ethics framework that offers a lens of understanding and heuristic guidelines to deal with ethical issues in citizen science.

**Methods:**

We conducted seven case studies between 2015 and 2021 to attune and validate the ethics framework for the context of citizen science. The cases related to studies with older adults, people with a psychiatric vulnerability, people dependent on community care, people who are unemployed or living in poverty or both, and young adults with respiratory disease.

**Results:**

Ethics in citizen science reaches beyond the ethical issues in traditional biomedical and health research. It entails more than following procedures about informed consent and privacy and submitting a proposal to a Medical Research Ethics Committee. Ethics in citizen science relates to everyday ethical issues during the study, including relational and moral complexities concerning collaboration, sharing power, and democratic decision-making. Dealing with these issues requires ethics work of researchers. This entails seeing ethically salient issues and reflecting on everyday ethical issues. Ethics work consists of seven features: framing work, role work, emotion work, identity work, reason work, relationship work, and performance work. All are relevant for researchers in citizen science.

**Conclusions:**

Ethical issues in citizen science often relate to power differentials, partnership, and collaboration between academics and non-academics. The ethics framework prepares researchers for the work needed in citizen science to act responsibly and offers a heuristic guide to reflect on ethics. Reflection on ethics is a pathway towards ethical citizen science, especially if researchers collaboratively reflect in partnership with non-academics who are subject to the moral issue.

## Background

Collaboration in scientific research with members of the public is increasingly encouraged and normalized. “Citizen science” [1, 2] has become an umbrella term that applies to a wide range of activities that involve the public in science. Citizens can become involved with scientific research in three distinct ways: (1) consultation; (2) collaboration; and, (3) control [3]. Firstly, consultation means inviting citizens’ opinions through surveys, for example, and could be a legitimate step toward their full participation, although it offers no assurance that citizens’ concerns and ideas will be taken into account. Secondly, in collaboration, power is in fact redistributed through deliberation and dialogue between citizens and researchers; it thus means to agree on full partnership and share planning and decision-making responsibilities. Finally, control means that citizens obtain the majority of decision-making seats, or full managerial power [4] in a study.

In full partnership model citizen science, members of the public whose lives are related to the topic of research share power and control equally throughout the research cycle in collaboration [5]. This model resembles participatory action research approaches for the co-creation of knowledge in dialogue and deliberation with citizens to improve their lives via collective action [6–9]. A new social contract between researchers, citizens, and government collaborating for the social good is at the core of citizen science and these related models. This field of research presents a growing area of opportunity for health and biomedical research to attune research better to the needs of the public, give citizens ‘a say,’ and mobilize the knowledge of the crowd to address complex problems.

In citizen science, members of the public are considered citizen co-researchers who bring different and complementary forms of knowledge, expertise, and skills. However, this new form of research surfaces diverse ethical issues that fall outside of and build upon standard human subjects’ concerns in bioethics [10]. Although the basic idea of this type of science is social inclusion [11], it is not apparent that citizens are heard in all phases of the study. Nor is it standard practice that members of the public receive appropriate funding for their activities, which is especially striking if the researchers get paid for their study. Another important issue that needs to be addressed is the respect, support, and facilitation of citizen researchers.

Ironically, despite researchers’ intention to create a space for the perspective of citizen researchers, in practice their voice might get silenced [12, 13]. Silencing may occur because of the hierarchy of knowledge, where scientific knowledge is perceived to stand above lay knowledge and where researchers keep positions of power. Moreover, academics work according to system guidelines or need to meet tight deadlines thereby often ignoring the capacities and needs of citizen co-researchers to participate [14, 15]. Silencing leads to “epistemic injustice,” a concept defined by Miranda Fricker [16, 17]. Epistemic injustice concerns a situation in which people are wronged in their capacity as knowers. It refers to situations in which citizen co-researchers feel that their capacity as knowers is undervalued and that their stories are not deemed worth being listened to by academics, especially in phases beyond data collection. This could have a negative effect on their motivation and involvement.

Thus, using the lens of epistemic (in)justice, new ethical issues emerge related to collaboration, co-ownership, and democratic decision-making. These new ethical issues are an addition to the important ethical issues in citizen science like dilemmas of data quality and integrity, data sharing and intellectual property, conflict of interest and exploitation [18]. For these ethical issues, principle-based approaches to ethics [19, 20] provide guidance. Besides, the International Collaboration of Participatory Health Research [21] developed ethical principles to complement these principles, from the perspective of participatory health research. These principles are: (1) mutual respect; (2) equality and inclusion; (3) democratic participation; (4) active learning; (5) making a difference; (6) collective action; and, (7) personal integrity. Although these principles are not yet standard for Medical Research Ethics Committees (MRECs), they are used regularly in the field of participatory action research [22].

However, in practice, despite the guidance of principles, researchers must work daily on ethical tensions to deal with the particular issue at that moment, in that specific context, taking into account the moral responsibilities to continue the research project [12, 23] from a commitment to epistemic justice. For this reason, this article presents a thematic analysis of the additional work that needs to be done by researchers regarding ethics in daily practice, besides the compliance work to meet standard principles. It offers an additional ethics framework for citizen science for daily practice.

## Methods

To explore ethical issues in citizen science and patient and public involvement (PPI) and to develop a specific framework for ethics in citizen science, we conducted seven case studies over 6 years (2015–2021) in the Netherlands. These case studies were grounded in participatory action research [7] and related to different groups of citizens: people who receive community care [24]; older people [25, 26]; children and parents in poverty [27]; people without a job who are dependent on social benefits [28]; families in a vulnerable situation [29]; people with experience of psychiatric crisis [30, 31]; and young adults with a respiratory disease [32]. The iterative multiple case study approach enabled us to work with members of the public and people with different backgrounds, diverse research questions and aims, and various settings.

The cases were not selected a priori, and the sample rather emerged on pragmatic grounds [cf. 33]. The authors coordinated the studies. In all cases, the project teams consisted of academics and citizens as co-researchers. The studies lasted between one-and-a-half and 5 years (Table 1).

**Table 1:** Overview of the seven projects involved in the multiple case study

Context	Citizens as co-researchers	Topic of the study	Period	Funding	Way of involvement
Building the Community of Practice	Experts-by- experience of care with community care	Partnership, power, and collaboration	2015–2021	Partner organizations of the CvC	Partnership
Age Friendly City	Older adults	Age-friendliness of a neighborhood	2016–2021	Municipality	Control
Health promotion	Children and parents in poverty	Reinforcing stigmas	2015–2019	Fonds Nuts Ohra	Partnership
Service delivery unemployment	People without a job, dependent on social benefits	Experiential knowledge of being unemployed	2017–2018	Municipality	Partnership
Funding organization	Families in a vulnerable situation	Engagement in a funding organization	2016–2021	Fonds Nuts Ohra	Consultation
Psychiatry emergency care	Experts-by- experience of care in psychiatric crisis	Improvement of emergency care	2016–2018	Care institution	Partnership
Academic medical hospital	Young adults with a respiratory disease	Improvement of clinical care setting	2018–2020	Dutch Foundation for Asthma Prevention	Consultation
Arts and Health	Older adults	Value of arts and health	2019–2021	ZonMw	Consultation

Most cases were embedded in the Centre of Client Experiences, CvC for short (www.centrumvoorclientervaringen.com), a platform that stimulates ethical reflection on projects with a participatory approach. This is a platform in which citizens, managers from care institutions, researchers, municipality officers, and patient advocacy organizations learn together in working sessions [15]. The Centre aims to improve the lives of people in vulnerable situations by participatory action research. Partners come from different settings in healthcare and well-being, including health providers, municipalities, research-funding or charitable organizations, and (applied) universities. Above all, a group of people with lived experiences were full partners in the CvC from the moment it was established. These people have daily experiences living in vulnerable situations and were eager to make a difference for themselves and others in a similar situation.

The authors systematically conducted reflection on ethical issues during the 7 years of this study. This was an iterative process [34] whereby the authors developed their understanding over time and refined their framework using concepts from the field of ethics, such as the notion of responsibility developed by care ethicist Joan Tronto [35]. Gradually the “ethics work” concept [36] was adopted as a lens for data analysis and as a sensitizing concept [34]. During the process, we developed and adjusted the framework to the context of citizen science. Finally, the first author also wrote her thesis on this topic [15].

There are various frameworks for research ethics, such as a rule-based or human rights approach. We work from a care ethical approach with a focus on the lived experiences in everyday situations. The underlying notion in care ethics is that ethically sound decisions cannot be universally defined. What is ethically right depends on the particularities of a situation, its complexities often entail a myriad of values and value-commitments. Value plurality and the involvement of various participants with their own perspective on a situation requires a detailed and in-depth understanding of all values and perspectives that matter in a specific situation to determine what is the best action for that case [37]. This implies that ethical challenges and tensions cannot be prevented and completely regulated by ethical guidelines and principles. It is the responsibility of researchers to be sensitive to and see ethically salient situations and act in an ethical way [22].

The notion of “ethics work” was developed in line with the above by Sarah Banks in social work [36]. Ethics work is a concept that focuses on the effort that one needs to put into an ethical issue to understand and decide what is the right course of action in that particular situation. It describes the ethical responsibilities and accompanying work to develop a responsible practice and become a responsible professional. Banks [36, p. 36] describes the concept as follows:

I am using the term ‘ethics work’ to refer to the effort people (in this case professionals) put into seeing ethically salient aspects of situations, developing themselves as good practitioners, working out the right course of action and justifying who they are and what they have done. Broadly speaking, ‘ethics’ relates to matters of harm and benefit, rights and responsibilities and good and bad qualities of character. I am using the term ‘work’ in this context to cover the psychological and bodily processes of noticing, attending, thinking, interacting and performing.

According to Banks [36], ethics work consists of seven features: framing work, role work, emotion work, identity work, reason work, relationship work, and performance work. Abma [14] suggests that ethics work and these features are embedded in the everyday practice of participatory action research. This article presents a framework for ethics in citizen science and other public and patient participation approaches that provides a tool for reflection during and after a study.

## Results

The framework for ethics in citizen science consists of seven types of work (Table 2). These features are presented in this section. Each type is illustrated with an example from the case studies. In this illustration, both the academic researcher and citizen co-researchers reflected on the ethical issues. It is a team arrangement to deal with ethical issues and collaboratively reflect on these everyday ethical issues. This is only possible if there is a communicative space in which people feel welcome and safe to learn together.

**Table 2:** Ethics work framework for citizen science, reframed from Banks [36]

Type of ethics work	Translation to ethical citizen science
Framing work	Being attentive to salient features of a situation and political listening (and viewing) Linking these features with structural mechanisms of marginalization Engaging in deliberations about these frames with citizen co-researchers and other stakeholders to co-create “new” frames
Role work	Playing a role in relation to others (researcher and researched, academic, and activist) and negotiating these roles Taking a position: sometimes being partial to the voice that is the least-heard, sometimes being impartial to being perceived as an academic
Emotion work	Being caring, compassionate, and empathic Building communicative spaces Seeing responsibilities of all involved in responding to others’ emotions
Identity work	Working that others see and experience the virtues of a caring ethical participatory researcher, for example towards the Medical Research Ethics Committees, funds, colleagues, citizens and clients Dialogue and deliberation about the ethos of a citizen science researcher and what ‘goodness’ means in relation to the people citizen science researchers work with
Reason work	Making decisions and justifying one’s decisions in ethically salient situations Conducting ethical reflections (individually and collaboratively) with those involved in the issue
Relationship work	Building trust and safety with attention to power relations and dependency so that everyone is seen, heard and valued Engaging in dialogue and deliberation with people, creating an open space for the experiences and perspectives of citizens Working on mutual, non-judgmental relationships through arts-based approaches, including representational knowledge
Performance work	Making visible aspects of this work to others Demonstrating oneself at work (accountability work)

### Framing work

Topics in citizen science are often political, and different perspectives on the topic are needed to come to an informed understanding and break through frames that marginalize people or repeat stereotypes. Framing work involves identifying and focusing on the ethically salient features of situations and placing oneself and the situations encountered in a political and social context [36]. In citizen science studies, this requires the academic researchers’ and involved others’ attentiveness to salient features of a situation.

For example, in a study with children in a poor neighborhood, the press was eager to listen politically to the researchers' stories and framed the study in the newspaper in a political light that re-established the notion that people are individually responsible for their behavior and situation. Instead, the researchers linked the study's insights to structural mechanisms of marginalization and engaged in deliberations about these frames with citizen co-researchers and other stakeholders. This work was complex and demanded creativity from the researchers to co-create 'new' frames with the children and their families. They countered the negative article in the newspaper by making their own newspaper with their frames. In this example, researchers made others aware of the context that shaped the situation of poverty.

An insight in this study is that framing work requires time, especially in polarized settings and concerning complex topics. Investing in understanding the various ways a situation can be framed—depending on one's role and position—respectfully and strategically by discussing which frames to use is often an ongoing activity during a citizen science study.

### Role work

All involved in citizen science—including academics, citizens, and other stakeholders—often perform several roles. This could be that of a researcher, but also of a facilitator, advocate, relation manager, or coffee-maker [38]. All roles are necessary to collaborate in a research team with different backgrounds with diverse needs. Role work includes identifying and performing one or more roles, negotiating roles, shifting between them, and sometimes taking a position in a situation (for example, close or distant) [36]. In citizen science, this means playing a role in relation to others (researcher and researched, academic and citizen) and being aware of these roles. Sometimes the least-heard needs to be encouraged and heard, for example. There are different expectations of academics but also citizens about roles in studies. For example, some do want to co-author academic articles to acknowledge their contribution to a study; others do not have any interest in co-authoring. They would like to be seen by the principal researcher and would like to be welcomed with pie for a meeting.

In a study on improving the emergency psychiatric ward, a director of the client advocacy organization in the city and cofounder of the platform Centre of Client Experiences could be seen as one of the citizen co-researchers working in a strategic position. At one specific moment at the start of the study into psychiatric emergency care, he practiced role work. The director deliberately took on the role of an advocate during a Town Hall meeting. He took a position on the voice that is the least-heard and used his position and power to raise interest in socially marginalized people. He did so in a diplomatic way, simply asking the question: “Did you ask the clients?” If he had not been in that room taking on the advocacy role, the question would not have been asked and the clients’ voices would have remained hidden and unheard, and thus the study would have been cancelled.

### Emotion work

When contexts of studies are loaded with emotions and citizen co-researchers and subjects are in a vulnerable situation, research teams need to put special effort into emotions. This could mean, for example, creating, nurturing and displaying emotions such as care, compassion, and empathy, but also exercising judgement about appropriate time and place for expressing, managing or suppressing emotions (such as distress, disgust, guilt, or fear) [36]. Academic researchers are not often used to working with emotions in their research practice, which traditionally promotes distance from subjects of studies. Besides, acknowledgment of the contribution of citizens in studies could also be emotion work. For some citizens, especially if they work voluntarily, a welcoming environment and positive energy could also be an acknowledgment. Emotion work requires the personal engagement of the research team members and their responsibility for caring relations between each other.

In a study about unemployment and living in poverty, the context and subject were associated with mainly negative emotions related to exclusion, health inequalities, and poverty. All citizen co-researchers were highly involved in the study. Emotion work consisted of the collaboration in the team and the responsibility everyone felt to care for each other’s well-being. For example, in group sessions, we learned that some of the citizens were perpetrators of child abuse, others were victims. The citizen co-researchers shared their impactful experiences about abuse in the group. The dynamic between these two core research groups of citizens was highly emotional and it was difficult for both parties to hear each other’s experiences. Dealing with the emotions of everyone involved felt like the researchers’ responsibility but also of all of the citizen co-researchers.

### Identity work

Working together with citizens, and especially citizens in vulnerable situations, demands an ethical ethos. This ethos is a personal matter; it requires working on one’s ethical self and virtue ethics [39]. Identity work consists of building, creating, negotiating, and maintaining an identity of a moral ‘good' professional. Besides the professional identity, this consists of being a ‘good' person, which requires personal, moral development (so-called Bildung). This is usually not part of the standard curriculum of a researcher, but the development of a personal, moral compass is and should be part of the training of citizen science researchers.

There is work to do to create an identity as an ethically good professional in traditional research institutions. For example, what is ‘goodness’ [40] concerning a citizen science researcher? From the ethics of care, values such as caring and relationships are essential, while from a more traditional ethical model, logic and reason are more critical virtues [35]. Schaffer [39] describes that compassion, courage, honesty, humility, justice, and practical reasoning are virtues and strengths of researchers that contribute to a life of flourishing or well-being for individuals and communities. Thus, concerning more traditional Ethics Committees, it is necessary to reflect on your professional, ethical identity: how you, as a researcher, will meet their standards and criteria and work inclusively, while maintaining professional integrity? Showing your virtues related to relational and care ethics and referring to the ethical principles, for example, the ICPHR principles [21] is essential to convince the members of these boards. If possible, an oral presentation and conversation can help clarify this and qualify oneself.

However, not only in preparation for a board meeting, identity work is essential. Often, research with citizens as partners is not “standard” in (medical) academic fields, so researchers continuously have to show that they work on their ethical self and are qualified as “good” researchers concerning people who live in vulnerable situations in conducting inclusive research. Training in virtue ethics and ‘goodness’ could help junior citizen science researchers prepare for this work.

### Reason work

Researchers may experience ethically salient situations daily. In all those moments, researchers must make and justify their decisions. Reason work consists of trying to see all sides of situations by considering all perspectives, all reasons for different pathways, and making ethical judgments. Therefore, it is essential to justify decisions by reasoning for actions and rehearsing ethical arguments [36].

In citizen science, a power hierarchy should be out of the question. However, there are always power differentials between people in the team. For example, in the Centre of Client Experiences we created room for ethical reflection together with all involved. There was a moment, for example, when a citizen co-researcher asked questions about power and who owned the project. It is an indication that the co-researcher felt safe enough to do so. Only then, a collaborative reflection could take place. This can only happen if academic researchers want to create room for such conversations. It can be an uncomfortable confrontation with one person’s privilege and another’s disadvantage. Moreover, it requires a willingness to reflect on one’s behavior and acknowledge that the previous approach was not the only “good” approach. Without listening, reflection, and action, citizen co-researchers might leave the building and the process. In a study with older adults, different citizen co-researchers stopped the study because they did not feel heard and did not see actions resulting from their deliberations.

It is not always possible to reflect collaboratively. A prerequisite is a safe space for all, a “communicative space” [41]. This is a space with a “good vibe,” in which openness and respect are central values. Engaging in a meaningful critical dialogical process is often impossible without a communicative space [7].

### Relationship work

Working with citizens and other stakeholders as partner in a study requires working with relations. Therefore, relationship work could be seen as work that flows from emotion, identity, and reason work, and thus be seen as dialogue work [36]. This could be function in relation to citizen co-researchers, as well as colleagues, officials, or other members of the public. An essential job in citizen science projects is building trust, being aware of the safety of the people involved, and being sensitive to power relations and dependency. This asks for attunement to the people and their needs to develop and contribute to a relationship where energy flows among people, because they feel seen, heard and appreciated, where everyone can give and receive without being judged, and wherein the relationship feels supportive and empowering [42]. In care ethics, trust is an important topic to engage people in the longer term. In our studies, we came across situations where co-researchers felt excluded, unsafe, or even violated in the process of citizen science [30].

In traditional verbal meetings, for instance, people who are less verbal are less able to talk or share experiences that matter to them. This asks for a creative dialogue in which arts-based approaches could help in conversations, relationship-building, and mutual understanding. For example, in a study about the value of active arts-engagement for older adults, it was difficult to engage with people with dementia. As a team with older adults as citizen co-researchers, we could not grasp the value of arts-engagement in words, but we would like to engage with these people and understand their value. This was important because officers of the government and health insurance provider wanted to hear about the value of arts as an intervention for health and well-being. A photographer, as a member of the researcher team, created a concept in which people were pictured before and after engaging in the art-activity. Making this series of photographs was a way to relate to the participants suffering from dementia, and moreover, it was a way to convey their experiences to third parties in a relatable manner. With dialogue sessions about these pictures, other stakeholders, like officers from the government empathized with the people with dementia and had a dialogue with them by discussing the pictures.

### Performance work

Finally, it is essential that peers and funders see that ethics work is a crucial and integral part of citizen science. If you strive for more inclusion and equality in research and academia, researchers must be willing to address ethical issues related to those core values. It is all too easy for these issues to be “brushed under the carpet” and show off with a version of citizen science that does not represent the reality and ignores “swampy questions” of the practice [43, 44]. Acknowledgement and transparency are important for the credibility of citizen science. Moreover, if funders do not see the importance of ethics work, researchers have to do this kind of invisible work in their own free time. Ethics is part of the quality of citizen science and should therefore receive dedicated attention from researchers and funding agencies, including appropriate funding.

## Discussion

This article shows that citizen science is much more than a technical endeavor in which academics apply suitable methods and techniques. It asks for ethical and normative work that scholars and funders can easily overlook. Ethics could easily be reduced to ticking a box on the checklist of the scientific board or Medical Research Ethics Committee. Researchers and co-researchers need, of course, be aware of the principle-based ethics and the ethical issues related to data quality and integrity, data sharing and intellectual property, conflict of interest and exploitation [18]. In a full partnership model, citizen science is a collaborative effort between citizens, researchers and other stakeholders. The research process aims to be democratic, participatory, empowering and educational. Therefore, the set of principles developed for participatory research [21] is useful as guidance in citizen science projects.

In practice, ethically complex situations may still arise. This implies ethically challenging situations in everyday research practice. There is ethics work to do on a daily basis during a process of citizen science, and this consists of framing, role, emotion, identity, reason, relationship, and performance work (Table 2). Ethics work is often invisible in citizen science if researchers are unaware of, or not open to, the complex moral issues encountered in relation to epistemic (in)justice [16, 17]. The framework of ethics work we have presented can be used as a heuristic: an approach that will help researchers and their teams be more sensitive and attend responsibly to daily ethical issues in citizen science. We recommend the use of the framework by research teams throughout a citizen science project as standard practice, besides more principle-based guidance focusing on participatory research, like ICPHR [21]. Team members should regularly discuss the various ethical principles, ethical dilemmas and types of ethics work they put into the project to ensure that this is not dependent on a specific situation or incident.

The ethics work that is an integral part of citizen science projects can get overlooked easily. We recommend that funding agencies provide appropriate resources to ensure that this type of work is carried out by one of the researchers. From PPI studies we know that this will be the most successful if the principal investigator is responsible for this type of work; if the principal investigator as team leader embodies the ethics work, this will have an impact on the entire research team. Too often such responsibilities are assigned to junior researchers or PhDs who are not in the position to create the conditions needed for optimal citizen involvement. We also recommend appointing an independent ‘citizen science’ critical friend to pose the more difficult and tough questions to the team.

The framework with the seven types of ethics work of citizen science can help to prevent that researchers associate ethics only with checklists and protocols. Ethics in citizen science is more than procedural work around Medical Research Ethics Committees and more than principles and regulations. Ethics work resonates with the citizen science literature, although this is a developing field. First, it relates to the concept of ethical boundary work, Kasperowski et al. [45] point out that ethical boundary work in citizen science is necessary. The authors refer to the boundaries between academics and non-academics, noting that researchers need to work at the boundaries to legitimate citizen science within academia (ibid.). These issues relate to framing work, role work, and reason work, as described in Table 2.

Secondly, the focus on relation and emotion work in citizen science literature is limited. For example, Fiske et al. [46] argue that more attention needs to be paid to the material and discursive exclusion of underserved populations in citizen science. Specifically, they argue that it would be reasonable to expect that meaningful participation and resulting benefits are equally distributed. Also, Smith et al. [47] propose to better value the various types of contributions of citizens' studies. They show that citizens often experience a lack of valuation of their contribution. The ethics work that needs to be done concerning acknowledgment of citizens in studies could vary in different types of citizen sciences. For example, in the full partnership model, the attention to emotion, relation, and identity work on acknowledgment will be more significant than in citizen science projects that resemble consultation or control models.

Thirdly, these scholars demonstrated that citizens often do not gain scientific, remunerative, or personal recognition from the beneficial outcomes of research applications. This underscores the inherent inequity, which may be seen as tokenism [47]. This also resonates in the work of Chesser et al. [48] who make a plea for, amongst others, sensitivity in studies that involve diverse and marginalized populations. The work that needs to be done to counter this lack of recognition is framing work, role work, emotion work, and relationship work. Therefore, it is vital to relate to citizens as partners, initiating dialogue about meaningful participation at the start of the study and maintaining it throughout. This can be different for every citizen that engages in a study.

Finally, as in the studies included in this study, ethical issues arise when individuals who have traditionally taken on the role of researcher or study subject can suddenly be both at the same time in citizen science. Having a dual role and identity, being researched and being a researcher, being an academic and a citizen may conflict with the traditional guidelines and procedures of Medical Research Ethics Committees [49]. Many experts-by-experience have felt embarrassed and insecure about revealing their identity [50, 51]. To deal with these different identities in studies requires role work of the researcher in practice, and performance work toward boards and funders that this type of work is necessary in studies.

Although the empirical data presented in this article is focused on different settings, with varying groups of citizens and gathered over an extended period with prolonged engagement [52], the limitation of this study is that the data is based on one country and one facilitating academic team. The experiences described in this article are mostly found in full partnership model citizen science in which people in vulnerable situations are involved in partnerships. In the field of citizen science, there is a group of scholars who work more in line with the consultation model wherein citizens join in to gather data rather than sharing power. Still, community- and activist-based citizen science initiatives focus more on epistemic justice and representation [45]. The insights in this study are relevant for all but may resonate more easily with the full partnership model citizen science academics.

This study showed the relevance of ethics work, which enriches ethics in citizen science. Ethics applies to work in the entire process. Developing ethical sensitivity is not something that researchers can learn from books; it is typically learned in a master-apprentice relationship. Like a craftsman, one learns over the years, from many cases, in close contact with the material and tangible reality of working with members of the public, what it means to do it well. This is certainly no simple task or responsibility. As sociologist Sennett points out, a craftsman “often faces conflicting objective standards of excellence; the desires to do something well for its own sake can be impaired by competitive pressure, by frustration, or obsession [53, p. 9].” Albeit not simple, it is also emotionally rewarding to do citizen science and develop pride in becoming a good citizen scientist.

## Conclusions

The moral-relational complexities of citizen science and the unique and challenging everyday ethical issues are not (yet) fully appreciated. This may lead to tokenism and instrumental engagement of citizens in citizen science projects. This study showed that ethics work needs to be done by the researcher to make citizen science studies inclusive and avoid epistemic injustice. Ethics work involves invisible work that is essential for meaningful participation and ethical citizen science. Scholars need to be aware of participatory greenwashing, which could be counterproductive for the trust of citizens in public engagement in science [7]. The ethics framework presented in this article could help researchers be aware of their responsibilities and make ethical boundary work accountable to ethics committees and funding agencies as well as to the larger public.

## Data Availability

Due to privacy, data are available from the authors upon reasonable request and with permission of the participants. Please email your request to the first author.
